# Changes in global quality of life after treatment with immune checkpoint inhibitors in patients receiving different treatment regimens for advanced stage lung cancer in the Netherlands: a 2015–2021 cohort study

**DOI:** 10.1136/bmjopen-2024-098062

**Published:** 2025-02-20

**Authors:** Ananya Malhotra, Erick Suazo-Zepeda, Petra C Vinke, Geertruida H de Bock, Willemijn J Maas, Jeroen T J.N Hiltermann, Bernard Rachet, Clémence Leyrat, Manuela Quaresma

**Affiliations:** 1 Health Services Research and Policy, London School of Hygiene & Tropical Medicine, London, England, UK; 2 Department of Epidemiology, University Medical Centre Groningen, Groningen, The Netherlands; 3 Department of Epidemiology, University of Groningen, Groningen, The Netherlands; 4 University Medical Centre Groningen, Groningen, The Netherlands; 5 Department of Neurology, Epidemiology, University Medical Centre Groningen, Groningen, The Netherlands; 6 Department of Pulmonology, University Medical Centre Groningen, Groningen, The Netherlands; 7 Health Services Research and Policy, London School of Hygiene and Tropical Medicine, London, UK; 8 Department of Medical Statistics, London School of Hygiene and Tropical Medicine, London, UK

**Keywords:** Quality of Life, Lung Neoplasms, EPIDEMIOLOGY

## Abstract

**Abstract:**

**Background:**

The introduction of immune checkpoint inhibitors (ICIs) has modified treatment modalities for patients with lung cancer, offering new alternatives for treatment. Despite improved survival benefits, ICIs may cause side effects impacting patients’ quality of life (QoL). We aim to study the changes in global QoL (gQoL) of patients with advanced-stage lung cancer up to 18 months after treatment with ICIs between 2015 and 2021.

**Methods and analysis:**

A longitudinal cohort study was conducted using the Oncological Life Study: Living well as a cancer survivor data-biobank from the University Medical Center Groningen. Participants completed the European Organisation for Research and Treatment of Cancer QoL 30-item questionnaire, at the beginning of their ICI treatment (baseline) and then at 6, 12 and 18 months. Using joint modelling, changes in predicted mean gQoL were studied by treatment regimens from baseline to 18 months, while accounting for the competing risk of death and adjusting for prespecified covariates.

**Results:**

Of the 418 participants with median age of 66 years, 39% were women. Patients receiving first-line immuno-monotherapy with palliative intent had a small improvement in their gQoL within 6 months and no clinically significant change thereafter. Patients receiving first-line immune-chemotherapy with palliative intent had a small improvement in their gQoL within 12 months and no clinically significant change thereafter. Patients with second/further line immunotherapy with palliative intent or first-line chemoradiotherapy followed by durvalumab with curative intent had no clinically significant change in their gQoL over 18 months.

**Conclusion:**

The changes in gQoL over time among patients with advanced-stage lung cancer may vary by treatment regimens based on drug intensity, line and intent of treatment, which will help clinicians and patients understand the potential dynamic of treatments on QoL. It may further influence treatment decisions and patient management strategies, reflecting the practical implications of different treatment regimens.

**STRENGTHS AND LIMITATIONS OF THIS STUDY**

- The study is based on a large real-world cohort of patients followed for 18 months after treatment with immune checkpoint inhibitors for their advanced-stage lung cancer.
- Missing data were handled using multiple imputation under a Bayesian framework, and the results were robust to deviations from a missing at random assumption.
- Selection bias may exist, as patients with higher baseline quality of life (QoL) were more likely to participate, and those with deteriorating health following treatment initiation were less likely to remain in the study.
- Average changes in QoL by cancer treatment regimens were studied rather than individual patient trajectories, so caution is needed when applying these findings to patient-level care in clinical settings.

## Introduction

 Lung cancer is the leading cause of cancer-related death worldwide (about 1.8 million (18%) deaths globally in 2020),^1^ largely because of a high proportion of advanced-stage (extent of primary tumour, condition of regional nodes and presence or absence of distant metastases stage 3 or 4) tumours with poor prognosis. The pattern is similar in the Netherlands, where advanced disease represents 49% of diagnosed cases, with 1-year overall survival at 46% for patients diagnosed between 2012 and 2018.^2^ Despite relatively recent advances in diagnosis and therapy, the prognosis for patients with advanced-stage lung cancer is still unsatisfactory. Since 2014, the introduction of immune checkpoint inhibitors (ICIs) has modified treatment modalities for patients with lung cancer, offering new alternatives for this disease in advanced stages.^3^ Despite its undeniable benefit in terms of survival,^4^ ICIs can cause side effects, which, in turn, may have an impact on the patient’s quality of life (QoL).^5^

In comparison with conventional therapies like chemotherapy, various studies report smaller impairments in health-related QoL scales, a longer time until deterioration in QoL and better control of symptoms after immunotherapy.^6^,^9^ This may be related to a lower risk of side effects from immunotherapy compared with chemotherapy.^10^ However, this evidence has largely come from clinical trials which have strict eligibility criteria; for example, these data exclude patients with poor performance status (PS, Eastern Cooperative Oncology Group PS>1), concomitant cancers or comorbidities. Hence, generalising the clinical benefits of ICIs seen in trial settings to real-world cohorts is hazardous.

Several population-based studies have revealed that patients’ socio-demographic characteristics, such as age, sex, education, as well as health status captured via PS, comorbidities or tumour stage, may impact their QoL.^11^,^14^ Hence, the effect of cancer treatment regimens on QoL outcomes may be confounded by these factors. Very few studies have been published on post-treatment, longer-term QoL based on observational real-world data, focusing on immunotherapy, as well as other cancer treatments such as chemotherapy and radiotherapy.^11^,^1315^ Moreover, studies that investigated QoL in this population so far had a follow-up period of less than 1 year,^16^ while ICI treatment regimens typically have an intended duration of 2 years.^17^ Patients’ QoL may, therefore, be affected even long after treatment with ICIs is initiated. The goals of therapy for advanced-stage lung cancer should not only focus on controlling the disease but also should be directed towards optimising the patient’s longer-term QoL. Our hypothesis is that for patients with advanced-stage lung cancer, the trajectory of their global QoL (gQoL) from baseline and over time varies according to the intensity, line and intent of the different treatment regimens, as well as their PS and comorbidities at baseline.

This research aims to study changes in the predicted gQoL of patients with advanced-stage lung cancer over 18 months after ICI initiation by different cancer treatment regimens in a real-world setting. We shall describe these changes in gQoL for patients with varying PS and comorbidities. The changes in predicted functional components of QoL, such as physical, emotional, social, role and cognitive, will also be studied in this population from ICI initiation up to 18 months. With this knowledge, clinicians and patients can be better informed as to what to expect in terms of the QoL of a patient after they receive immunotherapy.

## Methods

### Data source

This study was based on a subset of the OncoLifeS (Oncological Life Study: Living well as a cancer survivor) data,^18^ which is a hospital-based biobank of clinical well-being and QoL of patients with an oncological diagnosis and treated with anti-programmed cell D-1 (PD-1)/PD-1 ligand 1/cytotoxic T-lymphocyte-associated protein 4 ICIs at the University Medical Center Groningen (UMCG), the Netherlands. These data consist of linked routine clinical data, including cancer treatments, comorbidities, lifestyle, radiological and pathological findings, and side effects, with patient-reported data, including QoL, that are collected during and after their cancer treatment. Data on vital status was collected by linking the Dutch Basic Registration of Persons.^19^ OncoLifeS biobank was developed for oncological research with an overall aim to link routine clinical data with preserved biological specimens and QoL assessments (online supplemental appendix 1). It includes, among others, patients diagnosed with lung cancer and treated with ICIs from January 2015 onwards, who filled out questionnaires measuring their QoL around the time of ICI initiation and then every 6 months, up to 2 years. The data were pseudonymised by the project coordinator of OncoLifeS before analysis.^20^

### Inclusion criteria

The OncoLifeS database includes patients who were ≥18 years of age at the time of signing an informed consent. Patients receiving ICI treatment are those who have received one of the following monoclonal antibodies: nivolumab, pembrolizumab, cemiplimab, atezolizumab, avelumab or durvalumab.^21^ We included patients who were diagnosed with advanced-stage (3 or 4) lung cancer and treated with ICIs between January 2015 and November 2021, with no missing information on cancer treatments and who filled out at least one QoL questionnaire at baseline—either at the time of ICI initiation or up to 6 weeks before.

### Patient and public involvement

Patients and the public were not involved in the design, conduct, reporting or dissemination plans of this research.

### Outcomes and exposure

QoL scores were measured by the European Organisation for Research and Treatment of Cancer QoL’s^22^ 30-item questionnaire (EORTC QLQ-C30, V.3), which is part of an integrated system providing a QoL instrument to facilitate international clinical trials in oncology. These scores produce a continuous measure ranging from 0 to 100, with higher scores representing higher gQoL/higher level of functioning. Our primary outcome of interest was the gQoL score, and the secondary outcomes were the five functional scales (emotional, physical, social, role and cognitive) of QoL of patients diagnosed with advanced-stage lung cancer and treated with ICIs.

Patients were classified into either one of the four groups according to the line and intent of treatment (table 1). These groups reflect the intensity of the treatment regimens, and therefore, their possible implications on the patient’s QoL and the therapeutic intention (and indirectly on the patient’s prognosis).

**Table 1:** Criteria for classification of patients by their treatment regimen

Treatment regimens	Description	Line of treatment	Intent of treatment
Group 1	Chemoradiotherapy followed by durvalumab within 6 weeks to 3 months	First line	Curative
Group 2	Immuno-monotherapy	First line	Palliative
Group 3	Immuno-chemotherapy	First line	Palliative
Group 4*	Immunotherapy (as monotherapy or with chemotherapy)	Second or further line	Palliative

* Reference group, as a majority of patients in this cohort had immunotherapy as either second-line or further -line treatment, we wanted to compare this group to patients who had immunotherapy as a first -line treatment (groups 1, 2 and 3).

### Statistical analyses

The aim of this analysis was to study the changes in gQoL of patients with advanced-stage lung cancer, following their ICI treatment for up to 18 months, by different cancer treatment regimens. The repeated measurements of QoL scores enabled us to study how QoL changed over time. Box plots were used to describe the trajectories of continuous scores (ie, gQoL and the functional scales) from baseline to 18 months, conditional on patients’ survival at each follow-up time.

For assessing the longitudinal trajectory of QoL over time after ICI treatment, the competing risk of death was accounted for as it precludes patients from the outcome of interest, that is, QoL. Joint models^23^ were used to estimate the effect of treatment regimens on QoL over time following ICI treatment. Its two components allowed the simultaneous analysis of longitudinal and time-to-event data, which were linked using an association structure that quantifies the relationship between the change in QoL and survival. Due to the high number of patient drop-outs from either death or incomplete follow-up, we restricted our analysis to up to 18 months. Models were adjusted at the time of analysis for prespecified covariates, identified using a directed acyclic graph (DAG) created using DAGitty V.3.0^24^ (online supplemental file 2). Baseline covariables—age, sex, weight,^25 26^ education, PS, presence of concomitant cancer, presence of comorbidities (diabetes, hypertension, chronic obstructive pulmonary diseases, cardiovascular disease (CVD) and rheumatological conditions), number of months since lung cancer diagnosis and tumour stage—were identified as adjustment factors. ICI treatment response, side effects and ‘ICI stopping early’ were mediators in the path between treatment regimens and QoL, and hence not adjusted for in the analysis. The full model description is given in the online supplemental appendix 4.

Since a complete-case analysis may lead to biased results if data are not missing completely at random or if the missingness mechanism is not covariate-dependent only, we applied multiple imputation for handling missing data in the covariates—weight, education and PS—under a missing at random (MAR) mechanism, allowing for a possible association between missingness, treatment groups, covariates and outcome. Complex models such as joint models for longitudinal and survival data, in the presence of missing values, cannot be handled adequately by standard multiple imputation techniques. Using the *JointAI* R package,^27^ we fitted joint models using a fully Bayesian approach by modelling the analysis model (the joint model described above) jointly with the incomplete covariates under the MAR assumption,^28^ such that the analysis and imputation of missing data were performed simultaneously while ensuring compatibility between longitudinal and survival submodels. The full model description is given in the online supplemental appendix 5. The JointAI model gives a predicted QoL score, which is the mean of posterior distributions after 10 000 iterations, for each patient at each time point (0, 6, 12 and 18 months), along with a 95% prediction interval. For each patient, the difference between the posterior means of QoL score at two time points was computed, that is, between months 0–6, 6–12, 12–18, 0–12, 6–18 and 0–18. At each time point, the predicted mean gQoL score in a treatment group was computed by the average of the posterior means of the gQoL score of patients in the respective treatment group. The predicted mean of functional scores at a time point was computed by the average of the posterior means of functional scores at that time point. Change in QoL between two time points t1 and t2 (Δ_t1−t2_) was computed as the difference between the predicted mean QoL score at t1 and t2. Changes in QoL scores were interpreted as (1) an improvement in QoL for an increase in QoL score between two time points and (2) a deterioration in QoL for a decrease in QoL score between two time points. A minimal clinically important difference (MCID) in QoL between two time points was defined according to the evidence-based guidelines for interpreting changes in EORTC QLQ-C30 scores. For gQoL scores, a MCID between −5 and 5 points was defined as a trivial change, a MCID between −10 and −5 or 5 and 8 points was defined as a small deterioration or improvement, respectively, a MCID between −16 and −10 or >8 points was defined as a medium deterioration or improvement, respectively and a MCID<−16 was defined as a large deterioration in gQoL score (large improvement was not evaluable).^29^ These guidelines were developed by combining expert opinions and meta-analysis results from studies reporting QoL data using the EORTC QLQ-C30. While large, medium and small differences were defined as those with unequivocal, probable and subtle clinical relevance, respectively, trivial differences were defined as those likely to lack clinical relevance. Similar guidelines for thresholds defining improvement/deterioration in functional components of QoL have been used in this study for the interpretation of changes in these scores.

### Hypotheses

We hypothesise that the changes in the gQoL of patients following immunotherapy vary according to characteristics of the different treatment regimens (table 1) and patients’ health status: more intense regimens would be associated with a more pronounced initial decline in QoL, and lower PS and comorbidities would be associated with poorer baseline QoL, with minimal changes over time.

### Sensitivity analyses

We performed a sensitivity analysis by imputing missing observations on education under two extreme missing not at random (MNAR) mechanisms (ie, missing information related to non-measured data), assuming in turn that all the patients with missing education had a low level of education and then assuming they had a high level of education. We then compared the predicted trajectory of gQoL with the results obtained using multiple imputation above. We did not conduct such a sensitivity for weight and PS, as the proportion of missing data on these variables was very low.

All analyses were performed using R software V.4.3.0.^30^

## Results

There were 508 patients diagnosed with lung cancer between 1987 and 2021 and treated with ICIs between 2015 and 2021 at UMCG. Among these, 418 (82%) patients were included in our analysis because they filled out at least one QoL questionnaire and were diagnosed with stage 3 or 4 lung cancer. A consort diagram was used to describe the timeline of filling out EORTC QLQ-C30 (online supplemental appendix 2). Patients’ characteristics at baseline are presented in table 2 by treatment groups. The median age of patients at baseline was 66 years (Q1=59, Q3=71), and 161 (39%) were women. The majority of the patients (n=262, 63%) had second/further-line immunotherapy (group 4). Most patients had stage 4 lung cancer (n=369, 88%), about half of the patients were restricted in physically strenuous activity (PS=1, n=204, 49%), while 38% had a history of CVD (n=158), 58% had a low level of education (n=243). Over half of the patients (53%) reported side effects, and 21% reported severe (grade 3 or higher) side effects (table 2). There were higher proportions of patients in groups 1 and 3 with side effects (68% and 82%, respectively), but only 11% and 29% had severe side effects, respectively. Moreover, the majority of patients in groups 1 and 3 had either progressive disease or a partial response to treatment (table 2). Although less than half of the patients in groups 2 and 4 had side effects, the majority had progressive disease (61% and 73%, respectively). Observed QoL scores at baseline and follow-up times are presented in online supplemental figures A.3.1–A.3.6, appendix 3.

**Table 2:** Baseline clinical and demographic characteristics of patients with lung cancer by treatment regimen

Variable	Overall	Treatment regimen			
		Group 1: chemoradiotherapy followed by durvalumab	Group 2: immuno-monotherapy	Group 3: immuno-chemotherapy	Group 4: immunotherapy
		1st line	1st line	1st line	2nd/further line
		Curative intent	Palliative intent	Palliative intent	Palliative intent
Sample size (n, %)	418	37 (8.9)	70 (16.7)	49 (11.7)	262 (62.7)
Females (n, %)	161 (38.5)	13 (35.1)	25 (35.7)	14 (28.6)	109 (41.6)
Age at ICI treatment (years)					
Mean (SD)	65.12 (9.03)	63.70 (9.52)	65.07 (9.78)	67.47 (8.11)	64.90 (8.89)
Median (Q1, Q3)	66 (59, 71)	65 (61, 71)	66 (59, 73)	70 (62, 73)	66 (58, 71)
Weight (kg)					
Mean (SD)	78.78(15.96)	81.26 (15.51)	81.32 (18.76)	82.52 (17.78)	77.13 (14.73)
Median (Q1, Q3)	77 (67, 90)	81 (71, 93)	80 (69, 94)	82 (73, 93)	75 (67, 87)
Missing (n, %)	11 (3)	0 (0)	4 (6)	4 (8)	3 (1)
PS (n, %)					
No limit to normal activity: 0	182 (43.5)	26 (70.3)	26 (37.1)	20 (40.8)	110 (42.0)
Ambulatory, but restricted in strenuous activity: 1	204 (48.8)	11 (29.7)	35 (50.0)	25 (51.0)	133 (50.8)
Able to perform self-care, active>50% of daytime: 2	22 (5.3)	0 (0.0)	7 (10.0)	2 (4.1)	13 (5.0)
Only partly able to perform self-care, resting>50% of daytime: 3	4 (1.0)	0 (0.0)	1 (1.4)	0 (0.0)	3 (1.1)
Missing	6 (1.4)	0 (0.0)	1 (1.4)	2 (4.1)	3 (1.1)
Comorbidities					
Diabetes (n, %)	67 (16.0)	8 (21.6)	13 (18.6)	11 (22.4)	35 (13.4)
Hypertension (n, %)	182 (43.5)	20 (54.1)	32 (45.7)	26 (53.1)	104 (39.7)
COPD (n, %)	119 (28.5)	11 (29.7)	19 (27.1)	12 (24.5)	77 (29.4)
Rheumatological conditions (n, %)	37 (8.9)	5 (13.5)	11 (15.7)	3 (6.1)	18 (6.9)
History of CVD (n, %)	158 (37.8)	18 (48.6)	27 (38.6)	24 (49.0)	89 (34.0)
Concomitant cancer* (n, %)	34 (8.1)	1 (2.7)	6 (8.6)	6 (12.2)	21 (8.0)
Education (n, %)					
Low	243 (58.1)	21 (56.8)	39 (55.7)	28 (57.1)	155 (59.2)
Medium	71 (17.0)	8 (21.6)	12 (17.1)	8 (16.3)	43 (16.4)
High	75 (17.9)	7 (18.9)	11 (15.7)	12 (24.5)	45 (17.2)
Missing	29 (6.9)	1 (2.7)	8 (11.4)	1 (2.0)	19 (7.3)
Number of months since lung cancer diagnosis					
Mean (SD)	6.64 (8.20)	4.43 (2.29)	2.69 (4.94)	1.39 (3.04)	8.99 (9.11)
Median (Q1, Q3)	4 (1, 10)	4 (3, 5)	2 (1, 2)	1 (0, 2)	7 (2, 13)
Lung cancer stage (n, %)					
3	49 (11.7)	36 (97.3)	4† (5.7)	2‡ (4.1)	7§ (2.7)
4	369 (88.3)	1 (2.7)	66 (94.3)	47 (95.9)	255 (97.3)
Side effects (n, %)	220 (52.6)	25 (67.6)	34 (48.6)	40 (81.6)	121 (46.2)
Severe¶ side effects (n, %)	90 (21.5)	4 (10.8)	12 (17.1)	14 (28.6)	60 (22.9)
Treatment response (n, %)					
Complete response	20 (4.8)	4 (10.8)	4 (5.8)	0 (0.0)	12 (4.6)
Partial response	58 (13.9)	15 (40.6)	15 (21.4)	10 (20.4)	18 (6.9)
Stable disease	44 (10.5)	4 (10.8)	7 (10.0)	5 (10.2)	28 (10.7)
Progressive disease	281 (67.2)	14 (37.8)	43 (61.4)	32 (65.3)	192 (73.3)
Missing	15 (3.6)	0 (0.0)	1 (1.4)	2 (4.1)	12 (4.5)

* cConcomitant cancers include skin, breast, colorectal, bladder, prostrate, oropharynx, anorectal, esophagusoesophagus, lymphoma, endometrium, myelodysplastic syndrome, pancreatic, and vulva carcinoma.

† Tumour too large for curative radiotherapy or disease progression after chemo-radiotherapy after 6 months.

‡ rRecurrence after cT2bN3M0 treated with curative intent after 6 months or recurrence after cT4N0M0 treated with radiotherapy with curative intent after 6 months.

§ rRecurrence after chemo-radiotherapy before 6 months or cT4N3M0 and progression after palliative chemotherapy or progression after first -line chemotherapy (carbo-pemetrexed), fields too large for radiotherapy or recurrence cT4N0M0 after chemo-radiotherapy, carboplatin-gemcitabine, progression or carboplatin-gemcitabine for cT3N1MO0 no curative options.

¶ sSevere side- effects refer to grade 3 or higher side- effects as per Common Terminology Criteria for Adverse Events classification.

COPD, chronic obstructive pulmonary disease; CVD, cardiovascular disease; ICI, immune checkpoint inhibitorPSperformance statusQ1, first quartile; Q3, third quartileTNMT, extent of primary tumour; N condition of regional nodes and M, presence or absence of distant metastases

Predicted mean gQoL scores at baseline and follow-up time by different treatment regimen groups (table 3) show that patients in group 1 had the highest predicted mean gQoL (70.6, 95% prediction interval (60.1, 81.3)) at baseline compared with other groups. The trajectories of gQoL scores over 18 months after immunotherapy varied among patients’ different treatment regimens (online supplemental figure A.5.1, appendix 5 and table 4). Patients with first-line chemoradiotherapy followed by durvalumab with curative intent (group 1) had no clinically relevant changes in their gQoL from baseline to 18 months (difference between predicted mean gQoL score at baseline and 18 months (Δ_0−18_=1.4)). Patients with first-line immuno-monotherapy with palliative intent (group 2) had a small improvement in their gQoL within the first 6 months (Δ_0−6_=+6.3) and no clinically significant changes thereafter (Δ_6−18_=−3.9). Patients receiving first-line immuno-chemotherapy with a palliative intent (group 3) had a small improvement in their gQoL within the first 12 months (Δ_0−12_=+6.4) and no clinically significant changes thereafter (Δ_12−18_=+1.4). Patients in Group 4 with second/further-line immunotherapy with palliative intent had no clinically significant change in their gQoL from baseline to 18 months (Δ_0−18_=−3.7).

**Table 3:** Predicted mean QoL score with a 95% prediction interval by treatment regimens at baseline and follow-up times

	Month			
	0	6	12	18
Treatment regimen	Predicted mean gQoL score with a 95% prediction interval			
Group 1	70.6 (60.1, 81.3)	67.4 (57.1, 77.7)	67.9 (57.4, 78.4)	72.1 (60.3, 83.9)
Group 2	59.6 (49.9, 69.4)	65.9 (56.0, 75.8)	66.7 (56.5, 76.9)	62.0 (50.4, 73.7)
Group 3	58.4 (48.5, 68.3)	62.2 (51.9, 72.4)	64.8 (53.9, 75.7)	66.1 (51.3, 80.9)
Group 4	58.6 (50.4, 66.7)	58.6(50.4, 66.9)	57.4 (49.0, 65.7)	54.9 (46.1, 63.7)
Functional scale	Predicted mean functional QoL score with a 95% prediction interval			
Physical	70.7 (61.8, 79.7)	66.5 (57.6, 75.5)	64.4 (55.4, 73.5)	64.4 (55.1, 73.7)
Emotional	71.1 (62.4, 79.8)	75.3(66.5, 84)	76.5 (67.7, 85.3)	74.8 (65.7, 83.9)
Social	72.4 (62.2, 82.6)	70.9(60.7, 81.3)	69.6 (59.2, 80)	68.4 (57.6, 79)
Cognitive	83 (74.3, 91.8)	80.5(71.7, 89.3)	79.3 (70.4, 88.1)	79.4 (70.3, 88.5)
Role	60.6 (48.4, 72.7)	56.7(44.6, 68.9)	53.6 (41.3, 65.9)	51.1 (38.4, 63.8)

gQoLglobal quality of lifeQoLquality of life

**Table 4:** Changes in predicted mean gQoL scores from baseline to months 6, 12 and 18 by different treatment regimens

Treatment regimen	Difference between predicted mean gQoL score at baseline (month 0) and follow-up time (months 6, 12 and 18)								
	Month 0–6			Month 6–12			Month 12–18		
	Δ_0−6_	MCID	Clinical relevance	Δ_6−12_	MCID	Clinical relevance	Δ_12−18_	MCID	Clinical relevance
Group 1	−3.3	Trivial	No clinical relevance	+0.5	Trivial	No clinical relevance	+4.2	Trivial	No clinical relevance
Group 2	+6.3	Small improvement	Subtle	+0.8	Trivial	No clinical relevance	−4.7	Trivial	No clinical relevance
Group 3	+3.8	Trivial	No clinical relevance	+2.6	Trivial	No clinical relevance	+1.4	Trivial	No clinical relevance
Group 4	+0.1	Trivial	No clinical relevance	−1.2	Trivial	No clinical relevance	−2.5	Trivial	No clinical relevance

	Month 0–12			Month 6–18			Month 0–18		
	Δ_0−12_	MCID	Clinical relevance	Δ_6−18_	MCID	Clinical relevance	Δ_0−18_	MCID	Clinical relevance
Group 1	−2.8	Trivial	No clinical relevance	4.7	Trivial	No clinical relevance	1.4	Trivial	No clinical relevance
Group 2	7.1	Small improvement	Subtle	−3.9	Trivial	No clinical relevance	2.4	Small improvement	Subtle
Group 3	6.4	Small improvement	Subtle	3.9	Trivial	No clinical relevance	7.7	Small improvement	Subtle
Group 4	−1.2	Trivial	No clinical relevance	−3.7	Trivial	No clinical relevance	−3.7	Trivial	No clinical relevance

gQoLglobal quality of lifeMCIDminimal clinically important differenceΔchange in quality of life

**Figure 1:**
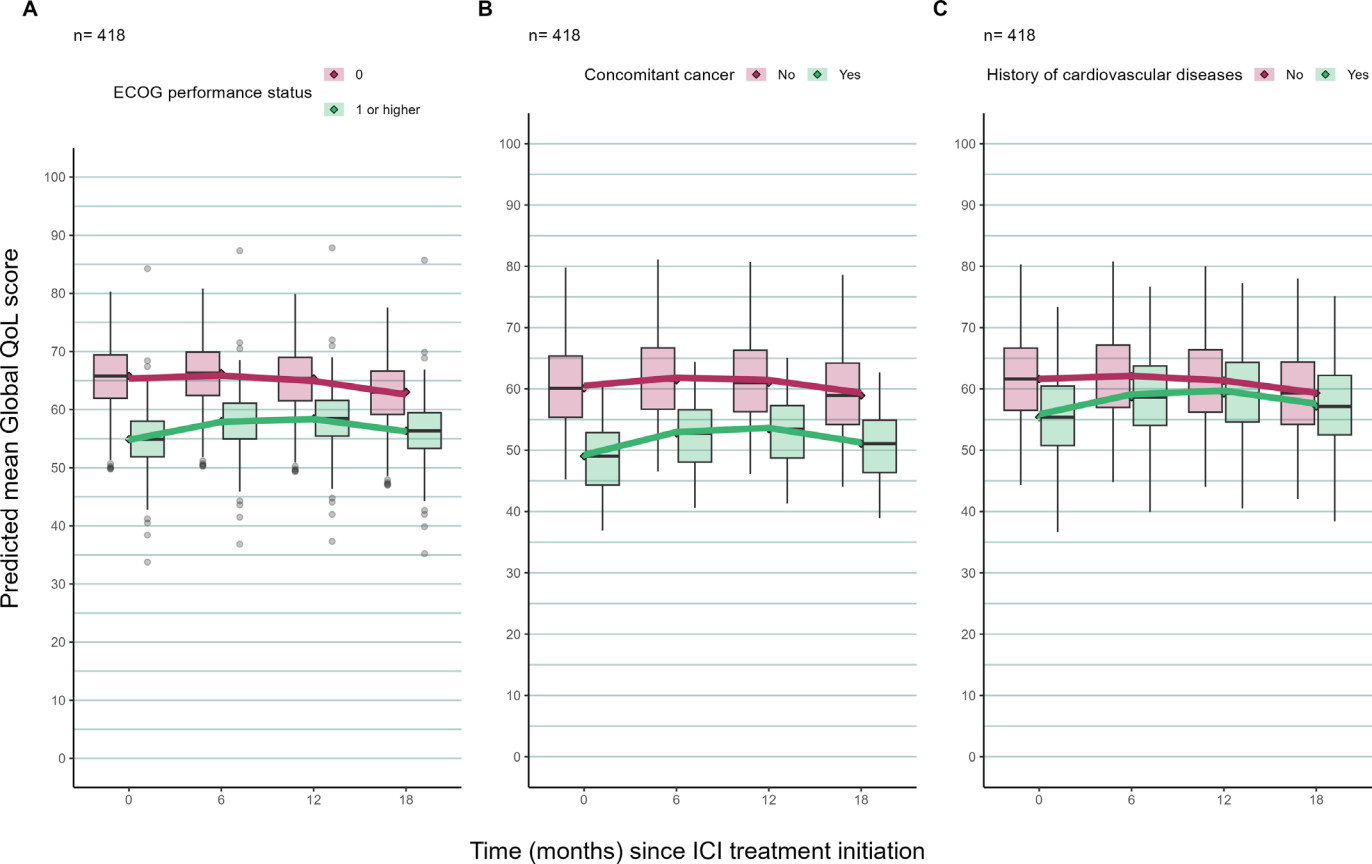
The predicted trajectory of global quality of life (gQoL) score of patients using a joint model with multiple imputation accounting for the competing risk of death by (A) performance status (PS), (B) concomitant cancer and (C) history of cardiovascular diseases. Box plots summarise the patient-level predicted mean gQoL score for each group. The superimposed curves show the predicted mean gQoL score in each group. Patients with PS=0 at baseline are those who are fully active; Patients with PS≥1 at baseline are those who are restricted in physical activity. ICI, immune checkpoint inhibitor and QoL, quality of life.

Variations in predicted mean gQoL trajectories by PS and comorbidities are described in figure 1. Patients with ‘fully active’ PS (=0) at baseline had higher predicted mean gQoL compared with those who were ambulatory but restricted in strenuous activity (PS≥1). However, there was no clinically relevant change in gQoL over 18 months in either of these subgroups. Patients with CVD or concomitant cancer had a lower gQoL compared with those who did not have these conditions; however, no clinically significant change in gQoL over time was predicted in either of these subgroups. Differences in predicted mean gQoL trajectories by other covariates were not clinically significant and hence not presented in this paper.

The results from the sensitivity analysis after imputing education under an MNAR mechanism (as ‘low’ or ‘high’) were similar to models fitted with multiple imputation (online supplemental figures A.5.2 and A.5.3, appendix 5), giving reassurance that our results were robust to departure from the MAR assumption assumed for multiple imputation.

**Figure 2:**
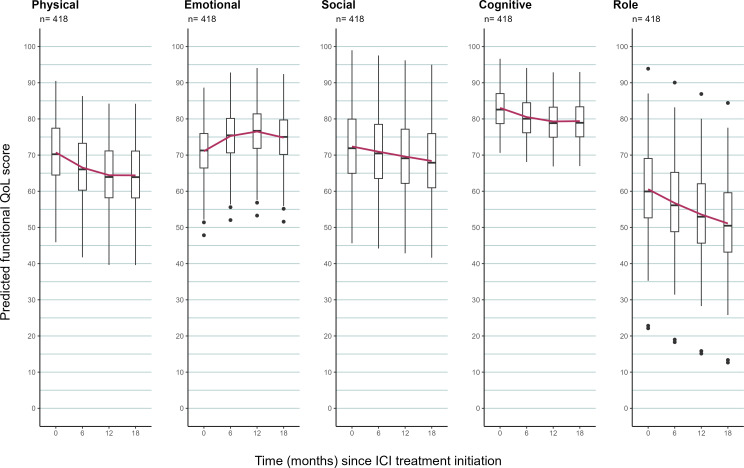
Predicted change in functional scale scores from a joint model with multiple imputation to account for missing data for the full cohort. Box plots summarise the individual-level predicted (adjusted) functional quality of life (QoL) score and are superimposed with a curve showing the population-level predicted (adjusted) functional score for the full cohort. ICI, immune checkpoint inhibitor.

Predicted mean functional scores at baseline and follow-up time (table 3) show that patients at baseline scored highest in cognitive functioning (83.0, 95% prediction interval (74.3, 91.8)) and least in role functioning (60.6, 95% prediction interval (48.4, 72.7)). The predicted mean trajectory of the five functional scores of patients with lung cancer from baseline to up to 18 months from the joint model with multiple imputation is shown in figure 2 and table 5. There was a small deterioration in physical function in the first 12 months after immunotherapy (Δ_0−12_=−6.3) and no clinically relevant change thereafter (Δ_12−18_=0). Emotional and social functioning had no clinically relevant changes from baseline to 18 months. Cognitive and role functioning had a small deterioration in the first 12 months after immunotherapy (Δ_0−12_=−3.7 and −7.0, respectively) and no clinically relevant change thereafter (Δ_12−18_=+0.1 and −2.5, respectively).

**Table 5:** Changes in predicted mean functional QoL scores from baseline to months 6, 12 and 18 by different treatment regimens

Functional scale	Difference between predicted mean functional QoL score at baseline (month 0) and follow-up time (months 6, 12 and 18)								
	Month 0–6			Month 6–12			Month 12–18		
	Δ_0−6_	MCID	Clinical relevance	Δ_6−12_	MCID	Clinical relevance	Δ_12−18_	MCID	Clinical relevance
Physical	−4.2	Trivial	No clinical relevance	−2.1	Trivial	No clinical relevance	0.0	Trivial	No clinical relevance
Emotional	+4.2	Trivial	No clinical relevance	+1.2	Trivial	No clinical relevance	−1.7	Trivial	No clinical relevance
Social	−1.4	Trivial	No clinical relevance	−1.3	Trivial	No clinical relevance	−1.2	Trivial	No clinical relevance
Cognitive	−2.6	Small deterioration	Subtle	−1.2	Small deterioration	Subtle	+0.1	Trivial	No clinical relevance
Role	−3.8	Trivial	No clinical relevance	−3.2	Trivial	No clinical relevance	−2.5	Trivial	No clinical relevance

Functional scale	Month 0–12			Month 6–18			Month 0–18		
	Δ_0−12_	MCID	Clinical relevance	Δ_6−18_	MCID	Clinical relevance	Δ_0−18_	MCID	Clinical relevance
Physical	−6.3	Small deterioration	Subtle	−2.1	Trivial	No clinical relevance	−6.3	Small deterioration	Subtle
Emotional	+5.4	Trivial	No clinical relevance	+3.7	Trivial	No clinical relevance	−0.4	Trivial	No clinical relevance
Social	−2.8	Trivial	No clinical relevance	−2.6	Trivial	No clinical relevance	−4.0	Trivial	No clinical relevance
Cognitive	−3.7	Small deterioration	Subtle	−1.1	Small deterioration	Subtle	−3.7	Small deterioration	Subtle
Role	−7.0	Small deterioration	Subtle	−5.6	Trivial	No clinical relevance	−9.5	Small deterioration	Subtle

MCIDminimal clinically important differenceQoLquality of lifeΔchange in quality of life

## Discussion

QoL indicators measured by patient-reported outcomes are pivotal in assessing the effect of cancer treatments on the QoL of patients with advanced-stage lung cancer. Such patients may experience a change in their QoL over time due to their prognosis and treatment-related side effects. Hence, research and clinical practice are ascending towards optimising patients’ QoL.^31^ The changes in QoL of lung cancer patients following ICI treatment have only received limited and recent attention in research,^32^,^34^ and our hypothesis was that trajectories of gQoL of patients over time vary by different treatment regimens. In this longitudinal cohort study, we aimed to study the changes in QoL of patients with advanced-stage lung cancer who were treated with ICIs and other cancer treatments at a tertiary cancer hospital in the Netherlands. Using joint modelling, which accounted for survival time and key confounders, we showed that there were differences in the trajectories of gQoL of patients according to treatment regimens based on its intensity, line and intent.

Patients who had treatment with curative intent (group 1) had a higher baseline gQoL than those with palliative treatments (groups 2–4), reflecting a less extensive disease (mainly stage 3) and/or better PS (generally=0). Since treatment intensity was maximal (chemoradiotherapy followed by durvalumab) among group 1 patients, a majority reported side effects, including a 10th with severe side effects. This explains a decline in the predicted mean gQoL score within the first 6 months of immunotherapy and then an improvement thereafter, although there was no clinically relevant change over 18 months. Overall, the presence or absence of chemotherapy in the treatment regimen seemed to be associated with transient deterioration (group 1) or improvement (group 2) of the gQoL, respectively. This agrees with previous studies that have shown ICIs to be associated with higher QoL and longer time to clinical deterioration compared with chemotherapy alone in different types of solid tumours.^32 33^ Moreover, in agreement with other studies^34 35^ where pembrolizumab (a PD-1 inhibitor) was given concurrently with chemotherapy, we found a small improvement in the gQoL of patients treated with immuno-chemotherapy (group 3), even if this improvement was initially slower than in the immuno-monotherapy group (group 2). This may also potentially be due to a larger proportion of group 3 patients reporting side effects compared with group 2.

QoL of patients with lung cancer treated with ICIs and pre-existing CVD is a complex and important consideration. Previous studies have shown that pre-existing CVD among patients with lung cancer treated with immunotherapy is associated with poorer overall survival.^36^ Our analysis showed that patients with lung cancer with a history of CVD had a lower gQoL over time (vs those with no CVD), which is a strong prognostic factor for survival in patients with non-small cell lung cancer (NSCLC).^37^ Since the incidence of clinically significant symptoms impacting QoL is greater among patients with advanced-stage lung cancer and poor PS,^38^ we also observed a lower gQoL over time among those with relatively poor PS (≥1) in our cohort. Patients with concomitant cancer within 1 year of ICI treatment initiation had a lower gQoL over time in our analysis; however, we did not find any evidence from the literature studying the impact of concomitant cancer on QoL. There was no association between age at ICI initiation and changes in gQoL in our study, which also supports the results of a recent study based on OncoLifeS data-biobank^14^ of patients with lung cancer treated with ICIs. A study on older patients with advanced lung cancer treated with systemic therapy reported a deterioration in physical functioning over a period of 6 months,^39^ which agrees with our results. Another study, in line with our results, reported a higher cognitive decline among patients with NSCLC treated with ICIs.^40^ Our analysis showed that there were no clinically significant changes in emotional and social functioning over time. This might be attributed to a response shift, which describes how patients psychologically adapt to changes in their health status over time.^41^

### Strengths and limitations

Our analyses were based on a large set of real-world data where patients were followed longitudinally over a period of 18 months after their ICI treatment. Linkage of these data with clinical records allowed us to adjust for important covariates. We have studied QoL using the EORTC QLQ-C30, a tool widely used in oncological research,^42^ which helped us to compare our results with similar studies. With repeated measurements on QoL, we developed a model that predicted the QoL trajectory of patients with advanced-stage lung cancer, from the start of their ICI treatment to up to 18 months. Exploration of the association between missingness and covariates, outcomes and treatment suggested some evidence of an outcome-dependent MAR mechanism. We addressed this issue of missing data in the covariates and performed a sensitivity analysis using multiple imputation under a Bayesian framework. We also checked the robustness of our results by imputing missing observations in ‘education’ under an MNAR mechanism and compared them with the results obtained using multiple imputation. Since the results were similar, we believe that our results were robust to some departure from the MAR assumption postulated for multiple imputation.

The UMCG, as a tertiary hospital, typically admits patients with more complex medical conditions and poorer survival prospects. From 2015 to 2021, lung cancer treatment underwent significant changes, particularly with the introduction of immunotherapy. By late 2018, guidelines recommended immuno-chemotherapy as the first-line treatment, altering the profile of patients in the OncoLifeS cohort. After the COVID-19 pandemic in 2019, secondary hospitals increasingly managed immunotherapy, leading to fewer referrals to tertiary care. As a result, from 2015 to 2018, our cohort mainly included patients receiving second-line monotherapy. After 2019, the cohort became more heterogeneous, with a likely more selective group of patients, often with more challenging clinical profiles and varied survival perspectives, many of whom received first-line combination therapies.^43^ These factors should be considered when interpreting and generalising our findings.

Specifically for lung cancer, the problem of incomplete questionnaires from dropouts or missing data from deceased patients during the observation period was inevitable.^44^ Hence, we could not perform our analysis on the full 2-year period, and instead had to restrict it to 18 months. There was a possibility for selection bias as patients with higher QoL were more likely to participate in the study than patients with lower QoL.^45^ If patients’ health deteriorated after treatment, their participation would have drastically reduced. We presented mean changes in QoL across subgroups of the populations defined by treatment regimens, rather than patient-specific trajectories of QoL. Therefore, our findings should be interpreted cautiously when applying them to patient-level care in clinical settings. We could not assess the interaction effect of treatment groups and PS on QoL due to the small numbers in these subgroups.

### Conclusion and recommendation for clinical practice

Our findings suggest that the trajectories of gQoL over time among patients with advanced-stage lung cancer may vary by treatment regimens based on drug combination, line and intent of treatment, which may help guide clinicians and patients on potential benefits and impairments of treatment regimens on QoL. This may further help identify patients who need additional care during their treatment. Since decision-making is largely driven by randomised trials, which do not provide a full picture because of their restricted inclusion criteria; hence, assessing the benefits of treatments through QoL measurements based on observational data is crucial.

## supplementary material

10.1136/bmjopen-2024-098062 online supplemental file 1

## Data Availability

Data are available upon reasonable request.
