# Supplementary material for: Changes in global quality of life after treatment with immune checkpoint inhibitors in patients receiving different treatment regimens for advanced stage lung cancer in the Netherlands: a 2015–2021 cohort study
Source: BMJ Open. 2025 Feb 20;15(2):e098062. doi: 10.1136/bmjopen-2024-098062 (PMC11843010; doi:10.1136/bmjopen-2024-098062)
Supplement: Supplementary file 2 [file bmjopen-2024-098062.reviewer_comments.pdf]

PEER REVIEW HISTORY

BMJ Open publishes all reviews undertaken for accepted manuscripts. Reviewers are asked to complete a checklist review form and are provided with free text boxes to elaborate on their assessment. These free text comments are reproduced below.

ARTICLE DETAILS

Title (Provisional)

Changes in Global Quality of Life after treatment with immune checkpoint inhibitors in patients receiving different treatment regimens for advanced stage lung cancer in the Netherlands – a 2015-2021 cohort study

Authors

Malhotra, Ananya; Suazo-Zepeda, Erick; Vinke, Petra C.; de Bock, Geertruida H.; Maas, Willemijn J; Hiltermann, Jeroen T.J.N.; Rachet, Bernard; Leyrat, Clémence; Quaresma, Manuela

---

VERSION 1 - REVIEW

**Reviewer**	**1**
**Name**	**Yano, Yoshitaka**
**Affiliation**	**Kyoto Pharmaceutical University, Education and Research**
**Date**	**21-Jan-2025**
**COI**	**None**

I have read the responses to the comments 2 and 3 and no more comments to them. However, I could not find the response to the comment 1 (The conclusion of this study ...) in the response letter, please let me know where the response, or add new response to the comment.

**Reviewer**	**2**
**Name**	**Kane, Eleanor**
**Affiliation**	**University of York, Health Sciences**
**Date**	**24-Jan-2025**
**COI**	**None**

Thank you for addressing my comments. On reflection, I have some more and please accept my apologies for not including these earlier. Hopefully these are straightforward and are intended to help with clarity:

1) Joint models were used to model the longitudinal gQoL while considering death. It is not stated where/how death information was collected in the study.

2) While I understand why the authors may want to examine the data in the way it is presented in Figure 1 (gQoL distributions by those surviving different periods), it is a presentation that takes into account a future event (death) and as such has issues of bias. It would be better not to present this (or if felt it is useful, that it is moved to the Supplementary Materials- is it already there in Figure A.3.1?).

3) If the above suggestion is made, the paper would present the distribution of gQoLs among the 4 treatment points as shown in Table 3 as its first main result which would be better.

4) The authors are suggesting in the response to comments including a box plot of the data shown in Table 3- both are not necessary and the data in Table 3 are a good description. The plot does include predicted trajectories from the joint model accounting for death- there doesn't appear to be much difference between the lines and where the medians are- if this is so, could this be stated rather than shown? If it is shown, consider improving the plot eg the lines are too thick.

**Reviewer**	**3**
**Name**	**Fukushima, Takuya**
**Affiliation**	**Kansai Medical University, Faculty of Rehabilitation**
**Date**	**26-Jan-2025**
**COI**	**None**

Thank you for giving me the opportunity to review this revised article.

I confirm that my comments have been fully addressed and reflected in the Results and Discussion section. I have no further comments.

VERSION 1 - AUTHOR RESPONSE

Reviewer: 1
Prof. Yoshitaka Yano, Kyoto Pharmaceutical University
Comments to the Author:
I have read the responses to the comments 2 and 3 and no more comments to them. However, I could not find the response to the comment 1 (The conclusion of this study ...) in the response letter, please let me know where the response, or add new response to the comment.
Apologies for not including our response here earlier. We had updated the conclusion of our findings from modelling results as well as clinical suggestions in the Conclusion section of the manuscript, and also stated below.

Conclusion and recommendation for clinical practice

"Our findings suggest that the trajectories of gQoL over time among advanced stage lung cancer patients may vary by treatment regimens based on drug combination, line and intent of treatment, which may help guide clinicians and patients of potential benefits and impairments of treatment regimens on QoL. This may further help identify patients who need additional care during their treatment. Since decision making is largely driven by randomised trials, which do not provide a full picture because of their restricted inclusion criteria, hence, assessing the benefits of treatments through QoL measurements based on observational data is crucial."

Reviewer: 2

Dr. Eleanor Kane, University of York

Comments to the Author:

Thank you for addressing my comments. On reflection, I have some more and please accept my apologies for not including these earlier. Hopefully these are straightforward and are intended to help with clarity:

1) Joint models were used to model the longitudinal gQoL while considering death. It is not stated where/how death information was collected in the study.

Information on death (vital status) was derived from the Dutch Basic Registration of Persons database which was linked to the OncoLifeS database. We have added this information in the manuscript under the Methods section, and also stated it below.

Methods

Data source

"This study was based on a subset of the OncoLifeS (Oncological Life Study: Living well as a cancer survivor) data,[18] which is a hospital-based biobank of clinical well-being and QoL of patients with an oncological diagnosis and treated with anti-programmed cell D-1(anti-PD-1)/ PD-1 ligand 1(anti-PD-L1)/ cytotoxic T-lymphocyte-associated protein 4 (CTLA-4) ICIs at the University Medical Center Groningen (UMCG), the Netherlands. These data consist of linked routine clinical data, including cancer treatments, comorbidities, lifestyle, radiological and pathological findings, side-effects with patient-reported data, including QoL, that are collected during and after their cancer treatment. Data on vital status was collected by linking the Dutch Basic Registration of Persons[19]."

2) While I understand why the authors may want to examine the data in the way it is presented in Figure 1 (gQoL distributions by those surviving different periods), it is a presentation that takes into account a future event (death) and as such has issues of bias. It would be better not to present this (or if felt it is useful, that it is moved to the Supplementary Materials- is it already there in Figure A.3.1?).

We have moved Figure 1 in the paper to the supplementary materials as Figure A.5.1.

3) If the above suggestion is made, the paper would present the distribution of gQoLs among the 4 treatment points as shown in Table 3 as its first main result which would be better.

Yes that is correct. Thank you.

4) The authors are suggesting in the response to comments including a box plot of the data shown in Table 3- both are not necessary and the data in Table 3 are a good description. The plot does include predicted trajectories from the joint model accounting for death- there doesn't appear to be much difference between the lines and where the medians are- if this is so, could this be stated

rather than shown? If it is shown, consider improving the plot eg the lines are too thick.

We agree that the data in Table 3 is a sufficient description of the main result. These results have also been stated in text in the manuscript under the Results section, and also stated below.
The plot (which was Figure 1 in the previous version and now moved to the appendix as Figure A.5.1 as per your suggestion above), has been improved by including thinner lines to illustrate trajectories of gQoL by different treatment groups.

Results
"Predicted mean gQoL scores at baseline and follow-up time by different treatment regimen groups (Table 3) show that group 1 patients had the highest predicted mean gQoL (70.6, 95% prediction interval [60.1, 81.3]) at baseline compared to other groups. The trajectories of gQoL scores of over 18 months after immunotherapy varied among patients different treatment regimens (Figure A.5.1 and Table 4). Patients with first-line chemo-radiotherapy followed by Durvalumab with curative intent (group 1) had no clinically relevant changes in their gQoL from baseline to 18 months (difference between predicted mean gQoL score at baseline and 18 months ($\Delta_{0\text{-}18}$=1.4). Patients with first-line immuno-monotherapy with palliative intent (group 2) had small improvement in their gQoL within first six months ($\Delta_{0\text{-}6}$=+6.3), and no clinically significant changes thereafter($\Delta_{6\text{-}18}$=-3.9). Patients receiving first-line immuno-chemotherapy with a palliative intent (group 3) had small improvement in their gQoL within first 12 months ($\Delta_{0\text{-}12}$=+6.4), and no clinically significant changes thereafter($\Delta_{12\text{-}18}$=+1.4). Group 4 patients with second/further-line immunotherapy with palliative intent had no clinically significant change in their gQoL from baseline to 18 months ($\Delta_{0\text{-}18}$=-3.7)."
"Predicted mean functional scores at baseline and follow-up time (Table 3) show that patients at baseline scored highest in cognitive functioning (83.0, 95% prediction interval [74.3, 91.8]) and least in role functioning (60.6, 95% prediction interval [48.4, 72.7]). The predicted mean trajectory of the five functional scores of lung cancer patients from baseline to up to 18 months from the joint model with multiple imputation is shown in Figure 2 and Table 5. There was a small deterioration in physical function in the first 12 months after immunotherapy ($\Delta_{0\text{-}12}$=-6.3) and no clinically relevant change thereafter ($\Delta_{12\text{-}18}$=0). Emotional and social functioning had no clinically relevant changes from baseline to 18 months. Cognitive and role functioning had a small deterioration in the first 12 months after immunotherapy ($\Delta_{0\text{-}12}$=-3.7 and -7.0 respectively) and no clinically relevant change thereafter ($\Delta_{12\text{-}18}$=+0.1 and -2.5 respectively)."

Reviewer: 3
Dr. Takuya Fukushima, Kansai Medical University
Comments to the Author:
Thank you for giving me the opportunity to review this revised article. I confirm that my comments have been fully addressed and reflected in the Results and Discussion section. I have no further comments.
